# Supplementary material for: Oxidative Stress Contributes to Bacterial Airborne Loss of Viability
Source: Microbiol Spectr. 2023 Mar 13;11(2):e03347-22. doi: 10.1128/spectrum.03347-22 (PMC10101003; doi:10.1128/spectrum.03347-22)
Supplement: Supplemental file 1 — Supplemental material. Download spectrum.03347-22-s0001.pdf, PDF file, 0.2 MB [file spectrum.03347-22-s0001.pdf]

Oxidative stress contributes to bacterial airborne loss of viability - Supplementary Information

Henry P. Oswin[1], Allen E. Haddrell[1], Cordelia Hughes[1], Mara Otero-Fernandez[1], Richard J. Thomas[2], Jonathan P. Reid[1]

[1]School of Chemistry, Cantock's Close, University of Bristol, Bristol BS8 1TS, United Kingdom

[2]Defence Science Technology Laboratory (DSTL), Porton Down, Salisbury, United Kingdom

SI Section 1

Improvements to methodology for CELEBS measurement of bacterial airborne viability

Since survival curves for *E. coli* MRE162 in airborne droplets of LB broth were published in 2020[1] several improvements have been made to the protocols used to make such measurements. This appears to have resulted in an improved survival at 50% RH in more recent measurements (compare Figure 1A from this study to Figure 3A in Fernandez 2020[1]). The changes made to the method that likely resulted in this improved survival will be described here.

Change to plating method: Initially a technique known as the 'pour plating' method was used to quantify the survival of bacteria from CELEBS levitations. In this method, the droplets were deposited directly into liquid LB broth, which was then left to sit for 5 minutes, allowing any aggregates to separate. Molten LB agar is then allowed to cool to 45°C before being added to the liquid medium containing the deposited droplets. The agar is then mixed into the liquid medium through a rotating motion. Once set, the plate is placed into a 37°C incubator overnight, allowing colonies to form from the bacteria embedded within the agar.

It was decided that the pour plating method was impractical for CELEBS measurements, with too much room for human error to influence results. If the agar was not allowed to sufficiently cool before adding it to the liquid media, the heat could kill some of the bacteria within the sample, reducing the observed survival from the measurement. Insufficient mixing of the agar and liquid medium can result in areas throughout which the bacteria can move more freely, resulting in areas of plate becoming confluent with bacterial growth preventing an accurate colony count.

Instead of pour plating, spread plating is now used for quantification of bacterial survival in airborne droplets. Rather than depositing into a petri dish entirely filled with liquid media, a small volume of media (typically 300µl) is placed onto the surface of solid media (typically LB agar for *E. coli* measurements). After deposition, the petri dish is left at room temperature for 5 minutes, again to allow disaggregation of bacteria and also dissolution of any solidified particles. A sterile L-shaped spreader is then used to gently spread the media containing the deposited droplets around the plate and the plate is placed into a 37°C incubator overnight.

Changes to data normalisation: Previously, data was normalised using a calibration curve[2] generated by spraying droplets with different CFU per ml onto a microscope slide and counting the number of bacteria within the droplet. In this way, the expected initial CFU per droplet for an experiment, to which the data from that experiment would be normalised, could be calculated by measuring the concentration of the culture loaded into the droplet dispenser and

using this calibration curve to find the resulting bacteria per droplet from that culture concentration.

However, there were several problems with this method of normalisation. Use of the calibration curve assumes that initial droplet sizes will remain consistent from one day to the next and will always be close to the size used to generate the curve. This isn't always the case, with initial droplet size being influenced by the dispenser used to generate the droplets, the viscosity of the solution forming the droplet, and the dimensions of the waveform sent to the piezoelectric component within the dispenser. Always using the same expected droplet size for normalisation will result in an increase in apparent survival for larger droplets and a decrease for smaller droplets.

It was possible to determine that for the majority of conditions studied, there was a lag before the viability of the bacteria began to drop in CELEBS, often taking 30 seconds to several minutes before lower than 100% survival could be consistently measured. Therefore, it was decided to use short levitations for normalisation instead of the calibration curve. For each different solution levitated, on each different day, several sub 5 second levitations are carried out and the average CFU per droplet from these levitations is used as our expected T0. Normalising to the average of these 5 second levitations, accounts for day-to-day variation in the size of the droplets and allows for a more accurate comparison of results from different experiments, and pooling of repeat measurements.

A comparison of survival measurements using both the old and new protocol to measure the decay of *E. coli* MRE162 at 70% RH is shown in Figure S1.

SI Section 2

Supplementary Figures

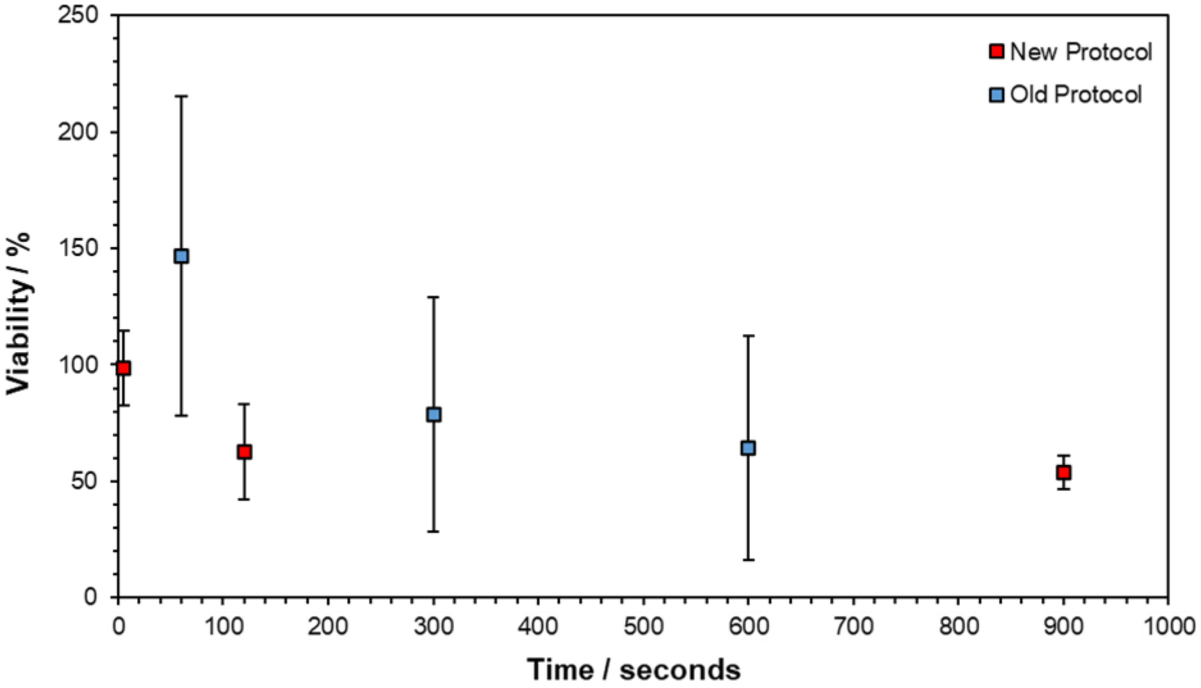

Figure S1| Comparison of the old and new protocols for measuring airborne bacterial viability in CELEBS. Levitations were carried out with E. coli MRE162 at 70% RH in LB broth droplets using both the old protocol (blue datapoints) and the new protocol (red datapoints). Datapoints show the average % viability and error bars show the standard deviation.

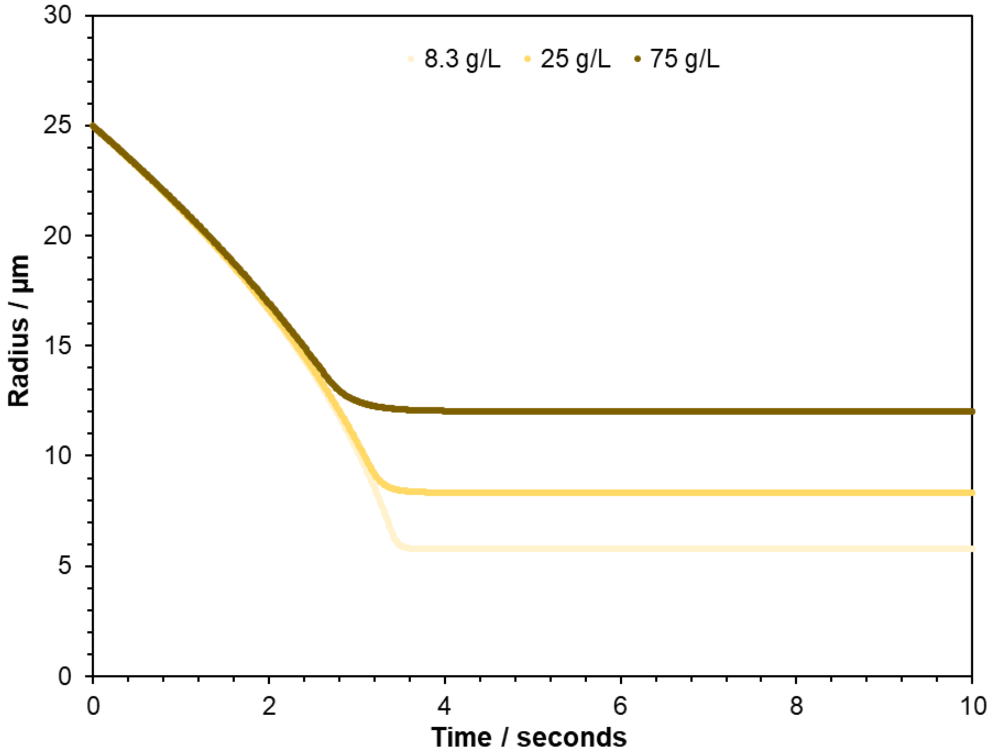

Figure S2| Impact of initial solute concentration on equilibrated size. Modelled size changes in LB broth with three different starting solute concentrations. Model uses data hygroscopicity data for LB broth previously collected using the CK-EDB.

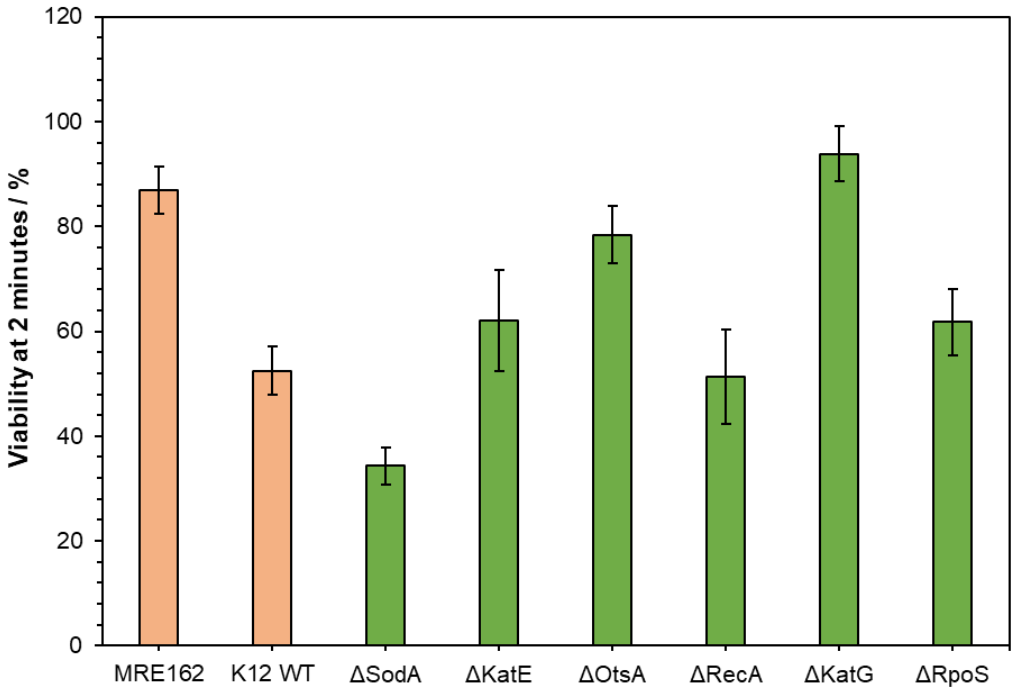

Figure S3| Airborne survival of *E. coli* K12 mutants. CELEBS measurements of the airborne survival of different *E. coli* deletion mutants after 2 minutes of levitation at 50% RH in droplets of LB broth. The bars show the mean survival, with the error bars showing the standard error.

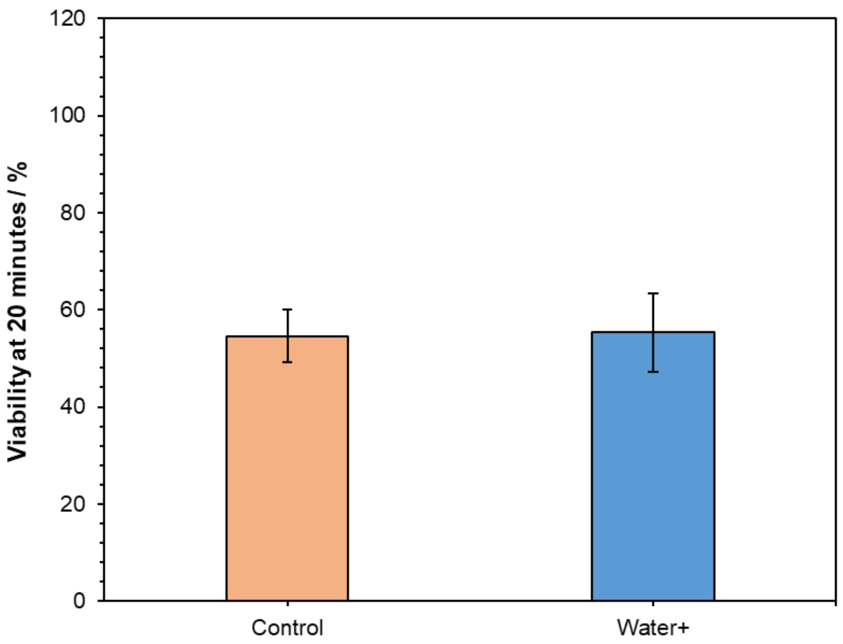

Figure S4| Control measurement for Antioxidant Experiments. *E. coli* MRE162 was levitated in LB broth at 50% RH, 18-21°C for 20 minutes. For the measurements shown in the blue bar, a small volume of water was added to the bacterial suspension prior to the CELEBS measurement, in the same way that a small volume of water containing various antioxidants was added to the suspension for the measurements shown in Figure 4C. This demonstrates that the improvement to survival shown in Fig. 4C was caused by the antioxidants and not by the slight dilution of LB broth that occurred when the antioxidants were added.

References

1. Fernandez, M. O., Thomas, R. J., Oswin, H., Haddrell, A. E. & Reid, J. P. Transformative Approach To Investigate the Microphysical Factors Influencing Airborne Transmission of Pathogens. *Appl. Environ. Microbiol.* **86**, e01543-20-e01543-20 (2020).

2. Fernandez, M. O. *et al.* Assessing the airborne survival of bacteria in populations of aerosol droplets with a novel technology. *J. R. Soc. Interface* **16**, 20180779 (2019).

3. Davis, E. J., Buehler, M. F., Ward, T. L., Davis, E. J. & Buehler, M. F. The double-ring electrodynamic balance for microparticle characterization. **1281**, (2017).

4. Bogan, M. J. & Agnes, G. R. MALDI-TOF-MS Analysis of Droplets Prepared in an Electrodynamic Balance : “ Wall-less ” Sample Preparation. **74**, 489–496 (2002).
